# Activation of the mitochondrial ATP-sensitive K^+^ channel reduces apoptosis of spleen mononuclear cells induced by hyperlipidemia

**DOI:** 10.1186/1476-511X-12-87

**Published:** 2013-06-14

**Authors:** Luciane C Alberici, Bruno A Paim, Karina G Zecchin, Sandra R Mirandola, Cezar R Pestana, Roger F Castilho, Anibal E Vercesi, Helena CF Oliveira

**Affiliations:** 1 Departamento de Patologia Clínica, Faculdade de Ciências Médicas, Universidade Estadual de Campinas (UNICAMP), Campinas, SP, Brazil; 2 Departamento de Física e Química, Faculdade de Ciências Farmacêuticas de Ribeirão Preto, Universidade de São Paulo (USP), Ribeirão Preto, SP, Brazil; 3 Departamento de Biologia Estrutural e Funcional, Instituto de Biologia, Universidade Estadual de Campinas (UNICAMP), Campinas, SP, Brazil

**Keywords:** Leukopenia, Hypertriglyceridemia, Mitochondria uncoupling, Cell redox state, Apoptosis

## Abstract

**Background:**

We have previously demonstrated that increased rates of superoxide generation by extra-mitochondrial enzymes induce the activation of the mitochondrial ATP-sensitive potassium channel (mitoK_ATP_) in the livers of hypertriglyceridemic (HTG) mice. The resulting mild uncoupling mediated by mitoK_ATP_ protects mitochondria against oxidative damage. In this study, we investigate whether immune cells from HTG mice also present increased mitoK_ATP_ activity and evaluate the influence of this trait on cell redox state and viability.

**Methods:**

Oxygen consumption (Clark-type electrode), reactive oxygen species production (dihydroethidium and H2-DCF-DA probes) and cell death (annexin V, cytocrome c release and Trypan blue exclusion) were determined in spleen mononuclear cells.

**Results:**

HTG mice mononuclear cells displayed increased mitoK_ATP_ activity, as evidenced by higher resting respiration rates that were sensitive to mitoK_ATP_ antagonists. Whole cell superoxide production and apoptosis rates were increased in HTG cells. Inhibition of mitoK_ATP_ further increased the production of reactive oxygen species and apoptosis in these cells. Incubation with HTG serum induced apoptosis more strongly in WT cells than in HTG mononuclear cells. Cytochrome c release into the cytosol and caspase 8 activity were both increased in HTG cells, indicating that cell death signaling starts upstream of the mitochondria but does involve this organelle. Accordingly, a reduced number of blood circulating lymphocytes was found in HTG mice.

**Conclusions:**

These results demonstrate that spleen mononuclear cells from hyperlipidemic mice have more active mitoK_ATP_ channels, which downregulate mitochondrial superoxide generation. The increased apoptosis rate observed in these cells is exacerbated by closing the mitoK_ATP_ channels. Thus, mitoK_ATP_ opening acts as a protective mechanism that reduces cell death induced by hyperlipidemia.

## Background

Circulating free fatty acids (FFA) may modulate immune cell functions when certain types are present at high levels. They can act either directly or by generating biologically active metabolites to regulate key metabolic and immune responses in lymphocytes, neutrophils and monocyte-macrophages. Fatty acids can modulate intracellular signaling pathways by changing cell membrane fluidity, the composition of lipid rafts and the production of second messengers. In addition, fatty acids can act on receptors in either the cell membrane or the nucleus, such as the toll-like receptors (TLRs) and the peroxisome-proliferator-activated receptors (PPARs), respectively. Common outcomes of fatty acid action on biological systems are stimulation of the production of eicosanoids, reactive oxygen species (ROS) and reactive nitrogen species, which may induce cell death [1].

Previous studies have demonstrated the induction of leukocyte death or proliferation suppression when these cells are exposed to high levels of FFA *in vitro* or obtained from animals fed high-fat diets. Several reports have indicated that fatty acids, particularly unsaturated fatty acids, can compromise leukocyte immune function, including cell proliferation, production of cytokines and natural killer cell activity [2,3]. However, the amount of fatty acids given in animal studies or the concentrations of FFA used in the cell culture studies often greatly exceed the amounts found in *in vivo* physiopathological conditions, which limits the biological significance of these results.

Transgenic mice overexpressing the apolipoprotein (apo) CIII exhibit markedly elevated plasma levels of triglycerides (TG), in addition to a ~2-fold increase in FFA levels, even on a very-low-fat diet [4,5]. Under normal laboratory controlled conditions, these mice are perfectly healthy. They present normal glucose homeostasis [6,7], as well as normal body mass and weight gain [8]. Therefore, these mice are useful models to study the effects of hyperlipidemia on cell function independently of secondary factors induced by high-fat diets, such as insulin resistance and obesity. Their hypertriglyceridemia is a consequence of the impaired liver removal of apo CIII-rich and TG-rich lipoproteins by their specific liver receptors [5]. Thus, the extended permanence of TG-rich lipoproteins in the plasma results in continuous FFA release to the plasma and tissues.

We have previously used these hypertriglyceridemic mice (HTG mice) to investigate the effects of hyperlipidemia on liver mitochondrial bioenergetics and redox state. We have found that liver mitochondria from HTG mice present increased resting respiration rates and reduced hydrogen peroxide release through a mechanism that is independent of uncoupling proteins or adenine nucleotide translocase activities and is related to the increased activity of mitochondrial ATP-sensitive K^+^ channels (mitoK_ATP_) [8-10]. This increased mitoK_ATP_ activity was also present in the brain but not the skeletal muscle of HTG mice [11]. MitoK_ATP_ activity results in a mild mitochondrial uncoupling that has little or no effect on oxidative phosphorylation efficiency [12]. Overall, this mitochondrial uncoupling process results in the increased consumption of substrates (including FFA), faster electron flow through respiratory chain complexes and less mitochondrial superoxide production [13,14]. We proposed that the increase in mitoK_ATP_ activity is a cell adaptation to reduce both intracellular FFA levels and mitochondrial superoxide generation [8].

This study was designed to investigate whether immune cells from HTG mice also present increased mitoK_ATP_ activity and how this activity influences the cell redox state and viability. Spleen mononuclear cells were chosen because they represent circulating blood lymphomononuclear cells and, in addition, these cells are relevant for atherosclerosis development in the hyperlipidemic context. Moreover, mitoK_ATP_ channels were already identified in a human T cell lymphoblast-like cell line (Jurkat cells) and display the main features of the mitoK_ATP_ channels found in the liver, e.g., they are blocked by ATP and selectively inhibited by 5-hydroxidecanoate [15]. We hypothesized that elevated TG and FFA levels induce an increase in mitoK_ATP_ activity, as observed in the liver, resulting in the protection of HTG mononuclear cells against oxidative stress and cell death.

## Results

**Figure 1:**
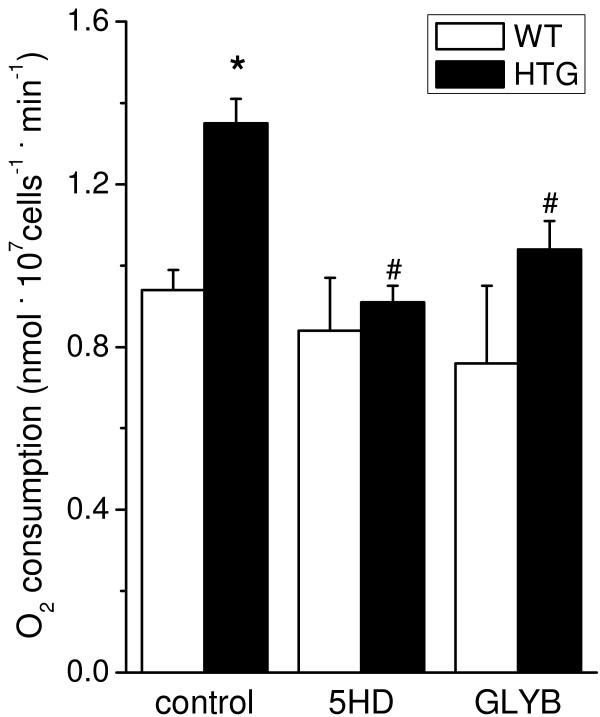
**Enhanced resting respiration in spleen mononuclear cells from HTG mice is due to the mitochondrial ATP**-**sensitive K**^**+ **^**channel (mitoK**_**ATP**_**).** HTG and WT cells (1.5 × 10^7^ cells.ml^-1^) were added to RPMI 1640 medium containing 25 mM HEPES pH 7.4, 1% FBS, 1 μg/ml oligomycin and 10 μM DPI, in the absence (control) or presence of 1 mM 5-hydroxidecanoate (5HD) or 10 μM glibenclamide (GLYB) at 37°C. The results are expressed as means ± SEM, n = 6. *P < 0.01 vs WT; ^#^P < 0.05 vs. HTG under control conditions.

Oxygen consumption by spleen mononuclear cells was measured under resting (non-phosphorylating) conditions to evaluate whether increased mitoK_ATP_ activity was present in HTG cells, as previously observed in liver mitochondria [8-10]. Figure 1 shows that oxygen consumption rates were approximately 40% higher in HTG cells than in WT cells under conditions where oxidative phosphorylation and NADPH oxidase were inhibited by oligomycin and diphenylene iodonium (DPI), respectively [16]. This increase in resting respiration was inhibited by two mitoK_ATP_ antagonists, i.e., glibenclamide (glyburide, GLYB) and 5-hydroxidecanoate (5-HD), indicating higher levels and/or activity of the mitoK_ATP_ channel in spleen mononuclear cells from HTG mice. No effects were observed in WT cells after treatment with mitoK_ATP_ antagonists, indicating low levels or activity of this channel in these cells.

**Figure 2:**
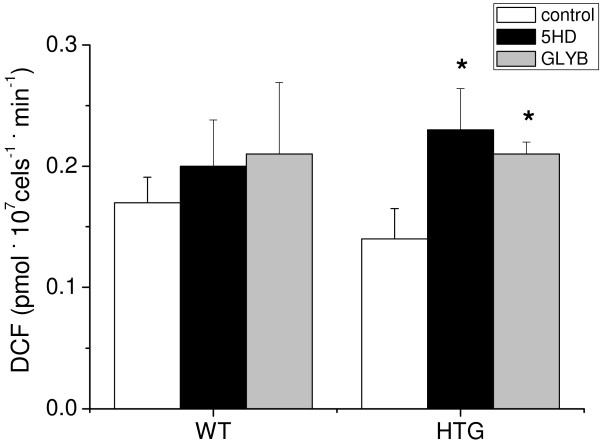
**MitoK**_**ATP **_**decreases mitochondrial ROS generation in spleen mononuclear cells from HTG mice.** HTG and WT cells (6 × 10^6^ cells.ml^-1^) were added to HANKS medium pH 7.4 containing 10 μM DPI and 1 μM H_2_DCF-DA in the absence (control) or presence of 1 mM 5HD or 10 μM glibenclamide (GLYB) at 37°C. The results are expressed as means ± SEM, n = 9. *P < 0.01 vs. HTG under control conditions.

In lymphocytes, NADPH oxidase and mitochondria are the main cellular sources of ROS generation [17,18]. Mitochondrial ROS production is modulated by respiratory rates. In general, faster respiratory rates, such as those found when uncoupling pathways are activated, are accompanied by lower levels of ROS release [13]. To specifically assess mitochondrial ROS production and its potential modulation via mitoK_ATP_ uncoupling [12], we conducted experiments in the presence of DPI to inhibit superoxide (O_2_^•-^) production via NADPH oxidase (control conditions). Under these conditions, using the membrane permeable non-specific probe H_2_-dichlorofluorescein-diacetate (H_2_DCF-DA), we found similar mitochondrial ROS production in HTG and WT intact mononuclear cells (Figure 2). However, when mitoK_ATP_ was closed by 5-HD or GLYB (Figure 2), increased mitochondrial ROS production was observed in HTG cells compared to the production in the respective control cells. These results suggest that high mitoK_ATP_ activity modulates mitochondrial ROS generation in intact spleen mononuclear cells from HTG mice.

**Figure 3:**
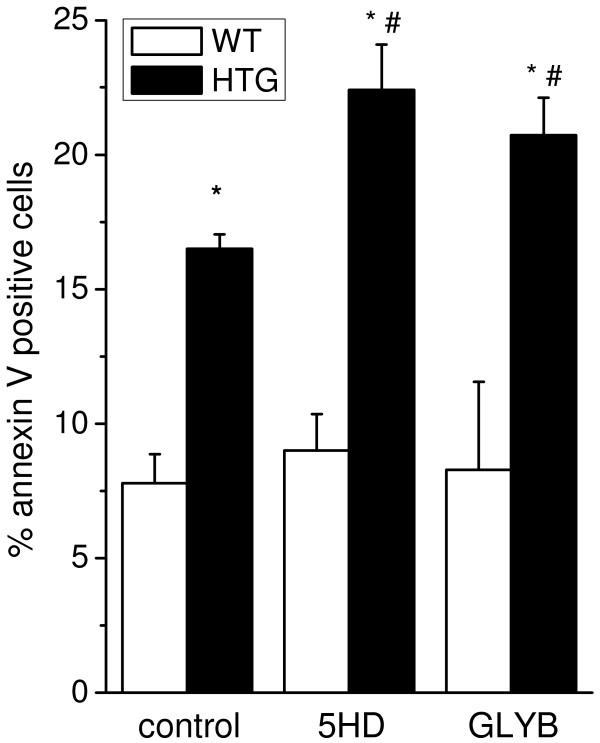
**MitoK**_**ATP **_**opening partially prevents enhanced apoptotic cell death in spleen mononuclear cells from HTG mice.** HTG and WT cells (10^6^ cells) were incubated for 20 min in binding buffer containing annexin V-FITC and 7-AAD, 10 μM DPI, at 37°C, in the absence (control) or presence of 1 mM 5HD or 10 μM GLYB, as described in the Materials and Methods Section. The results are expressed as means ± SEM, n = 5. *P < 0.01 vs. WT under same conditions; ^#^P < 0.05 vs. HTG under control conditions.

Next, we evaluated the role of increased mitoK_ATP_ activity on HTG mononuclear cell viability. The percentage of apoptotic cells was estimated using annexin V staining under the same conditions used to measure O_2_ consumption and ROS production. DPI was present to ensure that apoptosis pathways would not be induced by NADPH oxidase-generated ROS. Figure 3 shows that apoptosis levels were 2-fold higher in HTG than WT cells (control condition). Apoptosis in HTG cells was further increased in the presence of the mitoK_ATP_ antagonists 5-HD and GLYB (close to 3-fold higher in HTG than WT cells), while no changes in the apoptosis rates were observed in WT cells in the presence of these compounds. No significant levels of necrosis were detected in either type of cells (data not shown). These results indicate that increased mitoK_ATP_ activity partially prevents apoptotic cell death in HTG mononuclear cells.

**Figure 4:**
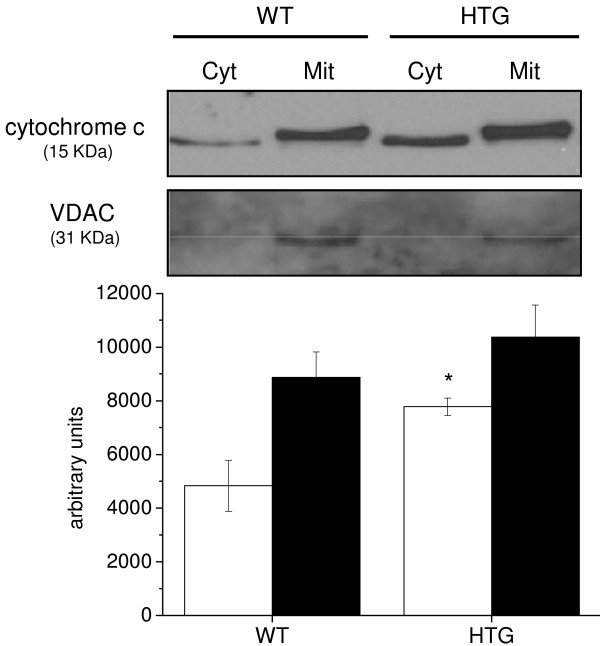
**Increased levels of cytochrome c in the cytosol of spleen mononuclear cells from HTG mice.** The cytochrome c content of mitochondrial (Mit) and cytosolic (Cyt) extracts were determined by Western blotting after 12% SDS-PAGE and staining with anti-cytochrome c antibody (~ 15 kDa), as described in the Materials and Methods Section. VDAC was used as the fraction purity control. The results are expressed as means ± SEM, n = 4. *P < 0.05 vs. WT.

Apoptosis can be induced by mitochondrial (intrinsic) and/or cell death receptor dependent (extrinsic) pathways, which can be respectively characterized by cytochrome c release and caspase 8 activation. We evaluated which of these pathways were involved in the apoptotic process in spleen mononuclear cells from HTG mice. Western blotting analyses of cell fractions showed larger quantities (~ 60%) of cytochrome c released into the cytosol of HTG cells in comparison with WT cells (Figure 4). In addition, we found approximately 70% higher caspase 8 activity in HTG cells (15.06 ± 2.19 pmol AFC. 10^6^ cells^-1^) than in WT cells (9.03 ± 1.3 pmol AFC. 10^6^ cells^-1^). These results indicate that apoptosis in HTG cells may be triggered by the extrinsic pathway upstream of the mitochondria but that these organelles are involved in the downstream stages of apoptosis.

The increased levels of apoptotic cell death of HTG cells could be related to the process of immune cell activation, which involves (i) the influx of extracellular Ca^2+^[17,19] and (ii) Ca^2+^-dependent activation of nuclear factors that upregulate genes encoding cytokines, such as IL-2, which is involved in cell cycle progression [20,21]. To test these possibilities, cytosolic free Ca^2+^ concentrations and IL-2 levels were measured in the spleen mononuclear cells of HTG mice. We found similar cytosolic free Ca^2+^ concentrations in both cell types (47.52 ± 2.45 and 50.46 ± 2.73 nM for WT and HTG, respectively) as well as very low but similar IL-2 levels in both cells (5.55 ± 1.62 and 5.85 ± 1.07 pmol.mg^-1^ for WT and HTG, respectively). These results indicate that the higher apoptosis levels in HTG cells were not related to the process of spleen mononuclear cell activation.

**Figure 5:**
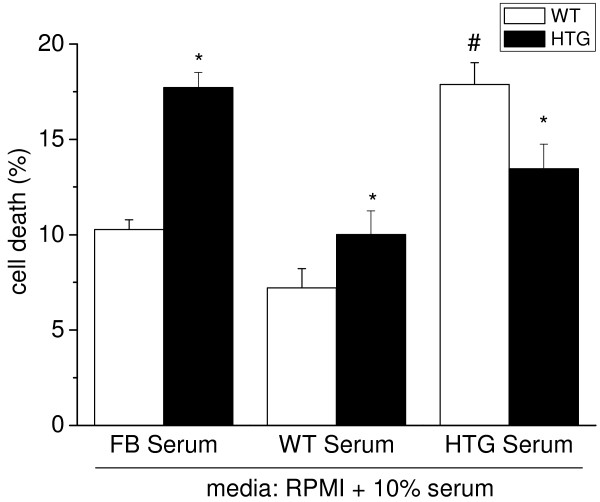
**Serum from HTG mice induces death in spleen mononuclear cells.** Cells (10^6^ cels.ml^-1^) were incubated for 4 h in RPMI 1640 medium containing 10% fetal bovine serum (FB Serum) or serum obtained from WT (WT Serum) or HTG (HTG Serum) mice. Cells were counted using a Neubauer chamber, using trypan blue to stain dead cells. The results are expressed as means ± SEM, n = 4. *P < 0.05 vs. WT in the same serum; ^#^P < 0.05 vs. WT in WT serum.

**Figure 6:**
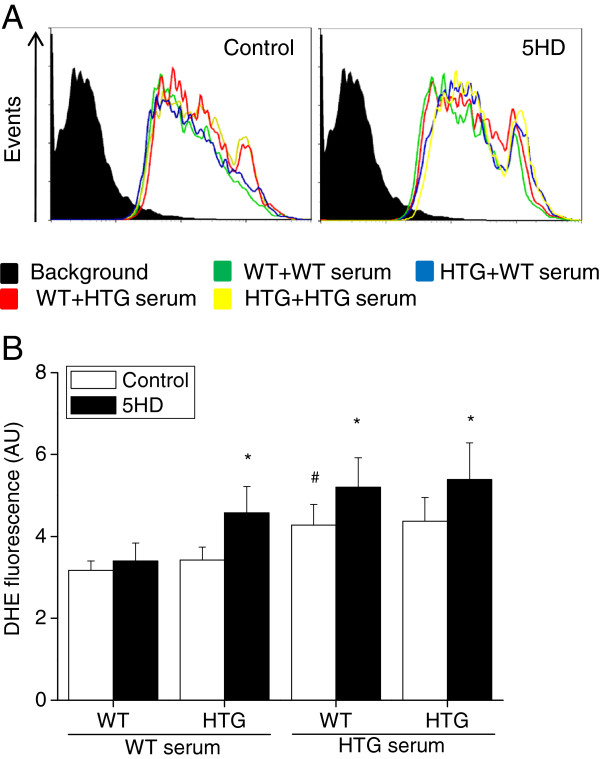
**Blood serum from HTG mice induces superoxide generation in spleen mononuclear cells.** Representative plots from flow cytometry analyses are shown in **A**, and means ± SEM are depicted in **B**. Cells (10^6^ cells.ml^-1^) were incubated for 4 h in RPMI 1640 medium containing 10% serum obtained from WT (WT Serum) or HTG (HTG Serum) mice. Green line: WT cells in WT serum; blue line: HTG cells in WT serum; red line: WT cells in HTG serum; and yellow line: HTG cells in HTG serum. *P < 0.05 for 5HD vs. respective control condition. The results are expressed as means ± SEM, n = 9. ^#^P < 0.05 vs. control WT cells in WT serum.

Previous studies suggested a significant relationship between peripheral human T cell anisotropy and plasma TG concentrations that was associated with the irreversible impairment of T cell proliferation and enhanced T cell apoptosis [22]. To evaluate whether the increased plasma lipid concentrations in HTG mice are the main inducers of cell death in spleen mononuclear cells, we compared cells incubated in media containing 10% fetal bovine serum or serum from WT or HTG mice (Figure 5). We found that, in the presence of normolipidemic serum (fetal bovine serum or WT serum), HTG cells presented higher cell death compared to WT cells (as observed in Figure 3). Conversely, when cells were incubated in the presence of the hyperlipidemic serum (HTG serum), we observed higher rates (74%) of death for WT cells than observed with normolipidemic serum. Under the same experimental conditions, hyperlipidemic serum elevated superoxide production by WT cells compared to normolipidemic serum (Figure 6). The presence of serum from HTG mice rendered the superoxide release in WT cells sensitive to the mitoK_ATP_ blocker 5HD, as observed in the HTG cells (Figure 6). These results indicate that components from HTG serum induce superoxide generation, activate mitoK_ATP_ and induce cell death in spleen mononuclear cells.

As the spleen is a site for the storage and rapid deployment of mononuclear cells [23], we investigated whether the enhanced susceptibility to apoptosis observed in the HTG spleen mononuclear cells could impact the levels of circulating mononuclear cells. In fact, we found that total white blood cell (WBC) counts in the peripheral blood were reduced by approximately 30% in HTG mice (Table 1). Among the main types of white cells, only lymphocyte counts showed a significant reduction in HTG peripheral blood compared to the level observed in WT blood. These results provide evidence of leukopenia in HTG mice.

**Table 1:** White blood cells (WBC) counts in blood from WT and HTG mice

	**WBC**	**neutrophils**	**lymphocytes**	**monocytes**
WT	6.7 ± 0.4	0.7 ± 0.1	5.8 ± 0.4	0.05 ± 0.02
HTG	4.8 ± 0.7*	0.7 ± 0.1	4.0 ± 0.7*	0.04 ± 0.03

Averages ± SEM, n = 8. Values are expressed in 10^3^ cells/mm^3^. *p < 0.05 vs WT.

## Discussion

The immune system cells have been shown to be impaired in several models of metabolic disorders, such as diabetes [24-26], obese-diabetic db/db mice [27], obese subjects [28], and genetically obese Zucker rats [29]. However, in these metabolic diseases, the co-existence of insulin resistance, disturbed lipid metabolism and chronic low grade inflammation impairs the determination of the relative contributors and the respective underlying mechanisms. In this regard, the HTG animal model studied here presents the advantage of a clean hyperlipidemia phenotype due to genetic manipulation.

In previous studies, we showed that the larger intracellular pool of FFA and TG in the livers of HTG mice was associated with increased mitochondrial resting respiratory rates due to enhanced mitoK_ATP_ but not UCP activity [8,9]. This condition resulted in higher tissue and whole body oxygen consumption [8] and less ROS generation by the mitochondria [10,11]. In the present study, we demonstrated that spleen mononuclear cells from HTG mice also presented higher mitoK_ATP_ channel activity, as the enhanced O_2_ consumption was sensitive to the mitoK_ATP_ antagonists 5HD and glibenclamide. As observed in the liver, mitoK_ATP_ activity also reduces mitochondrial superoxide production in HTG spleen mononuclear cells. A major finding of this work is that mitoK_ATP_ closure exacerbates apoptosis in these cells. Both extrinsic and intrinsic (mitochondrial) pathways seem to be involved in the apoptosis of HTG cells, as shown by caspase-8 activity and cytochrome c release to the cytosol. These two pathways could be connected through the cleavage and activation of the proapoptotic protein Bid, which is downstream of the caspase-8 step and upstream of the mitochondria [30,31]. Alternatively, both extrinsic and intrinsic pathways may occur independently and concurrently.

The serum reconstitution experiments detailed here show that components of HTG serum enhance apoptosis in WT cells. FFAs are believed to be the principal toxic trigger mediating the adverse cellular effects of lipids. In high concentrations, they are well known inducers of cell death in a variety of cell types. FFAs modulate the expression of several genes related to the cell cycle, apoptosis, proliferation, and oxidative stress in leukocytes in a dose- and type-dependent manner [32-36]. Several mechanisms of FFA-induced apoptosis have been proposed, mostly involving mitochondria, either directly [37-39] or at downstream steps of ROS-induced signaling pathways [38,40-44]. The cross-talk between mitochondria and both NADPH oxidase and xanthine oxidase [10,45] are of particular interest in the case of the HTG model because we have previously shown elevated activity of these two oxidases in the livers of HTG mice [10]. Considering that superoxide radicals and H_2_O_2_ can activate mitoK_ATP_ in vitro [46,47], we proposed that excess FFA activates the extra-mitochondrial oxidases and that the resulting superoxide radicals activate mitoK_ATP_, which in turn decreases mitochondrial superoxide generation. This proposal is fully supported by data that demonstrate that *in vivo* antioxidant treatment of HTG mice reversed whole liver oxidative damage, decreased mitoK_ATP_ opening and restored mitochondrial resting respiration [10]. Therefore, we have proposed mitoK_ATP_ as a redox sensor that allows for cross-talk between extra- and intra-mitochondrial compartments [10]. Accordingly, in the present work, we showed that serum from HTG mice induces superoxide radical production in WT cells, which is further upregulated by the mitoK_ATP_ blocker 5HD, indicating that hyperlipidemia induces ROS generation and activates mitoK_ATP_.

MitoK_ATP_ opening has previously been studied in the context of ischemia-reperfusion vascular damage. Several evidences have shown that pharmacological opening of mitoK_ATP_ protects against ischemic oxidative damage. However, the signals and mechanisms of activating the channel in vivo are still a matter of debate. Andrukhiv et al. [48] showed that when heart mitochondria were treated with the mitoK_ATP_ channel openers diazoxide or cromakalim, their ROS production increased by 40-50%, and this effect was blocked by 5-hydroxydecanoate. Thus, these authors propose that the mitoK_ATP_ leads to cardioprotection through an increase in ROS production. On the other hand, Facundo et al. [49] reported an enhancement of mitoK_ATP_ activity by treating heart mitochondria with H_2_O_2_ and an impairment of diazoxide-mediated activation of these channels by reducing agents. Thus, these authors propose that ROS production in reperfusion condition activates mitoK_ATP_ which then prevent further oxidative stress under this pathological condition. Accordingly, Queliconi et al. [7] showed that mitoK_ATP_ is directly activated by several (but not all) oxygen and nitrogen species in isolated heart mitochondria and that this redox modulation of mitoK_ATP_ may be the underlying mechanism for cardioprotection in ischemic preconditioning. Although the controversy about the effects of mitoK_ATP_ on ROS release remains [45,47-49], our previous and present data fit best in a view where mitoK_ATP_ activity represents a homeostatic mechanism that avoids amplifying the ROS production inside the cell [14,49,50].

FFA-induced apoptosis may be prevented by decreasing mitochondrial oxidative stress; for example, carnitine prevents FFA-induced apoptosis in HepG2 cells by increasing mitochondrial oxidation and reducing oxidative stress [51]. This is also confirmed here by the exacerbation of apoptosis after closing mitoK_ATP_ (Figure 3) and by the upregulation of ROS production upon mitoK_ATP_ blocking in WT and HTG cells exposed to HTG serum (Figure 6). In agreement with our results and proposal, nicorandil, a clinical drug used to treat ischemic heart disease, has been shown to protect cardiomyocytes from apoptosis by activating mitoK_ATP_ channels [52]. In addition, the nicorandil-induced opening of mitoK_ATP_ channels suppressed ROS-induced apoptosis in cultured cerebellar granule neurons [53].

Even when partially protected against apoptosis by mitoK_ATP_ activation, HTG mononuclear cells present higher rates of apoptosis under basal conditions. This finding may explain the lymphocytopenia observed in the blood of the HTG mice (30% reduction in total WBC). Interestingly, previous studies on human subjects showed a positive association between leukocyte counts and levels of circulating triglycerides [54,55]. However, in human epidemiological studies, the WBC count is commonly used as a surrogate marker of subclinical inflammation, a condition that is not present in HTG experimental model.

## Conclusions

Altogether, our results demonstrate that spleen mononuclear cells from hyperlipidemic HTG mice have an increased mitochondrial resting respiration rate due to increased activity of the mitoK_ATP_ channel, which downregulates mitochondrial superoxide generation. The increased levels of apoptosis in these cells are likely due to both intrinsic and extrinsic pathways. The levels of circulating lymphocytes are also reduced in HTG mice. We propose that serum FFA in HTG mice induces a cellular oxidized state that triggers apoptosis. The activation of mitoK_ATP_ is an adaptive response that increases FFA oxidation and decreases mitochondrial superoxide generation, which reduces the rates of the mitochondrial arm of the apoptosis pathway.

## Methods

### Animals

The human apolipoprotein CIII transgenic (line 3707) [56] founders were donated by Dr. Alan R. Tall (Columbia University, NY) in 1996 and bred in the animal facilities of the Department of Physiology and Biophysics, State University of Campinas, SP, Brazil. Experiments were approved by the University's Ethics Committee and are in accordance with the Guidelines for Handling and Training of Laboratory Animals published by the University’s Federation for Animal Welfare. Mice were housed at 22 ± 2°C on a 12-h light–dark cycle and had access to standard laboratory rodent chow (Nuvital CR1, Parana, Brazil) and water *ad libitum*. Male and female heterozygous apolipoprotein CIII transgenic (HTG) and non-transgenic (WT) littermates, aged 3 to 6 months, were used in this study. Transgenic mice presented fasting plasma triglyceride levels over 300 mg/dL and WT below 100 mg/dL. Plasma triglyceride levels were determined using an enzymatic-colorimetric method (Chod-Pap, Roche Diagnostic GmbH., Mannheim, Germany).

### Isolation of spleen mononuclear cells

Spleens were gently homogenized in a Douncer manual homogenizer. Spleen homogenates were overlaid onto a Ficoll-PaqueTM PLUS layer with the density adjusted to 1.076 g/mL and centrifuged at 1000 × *g* at room temperature for 25 min. The interface cell layer was recovered with a Pasteur pipette, washed twice in PBS and centrifuged at 500 × *g* for 10 min [57]. Cells were counted in a Neubauer chamber, using trypan blue to stain dead cells. Cells were used when the viability was > 98%.

### Determining oxygen consumption

Oxygen consumption was measured using a Clark-type electrode (Hansatech Instruments Limited, Norfolk, UK) in a 0.5 ml sealed glass cuvette equipped with a magnetic stirrer at 37°C. Reaction media for mitochondrial respiration consisted of RPMI 1640 medium, 25 mM HEPES pH 7.4, 1% FBS, 1 μg/ml oligomycin and 10 μM diphenyleneiodonium (DPI).

### Measuring intracellular reactive oxygen species (ROS) levels

Intracellular ROS generation was measured using 1 μM H_2_-dichlorofluorescein-diacetate (H_2_DCF-DA) [58] in a 1 mL cuvette with constant stirring at 37°C. Reaction media for measuring ROS generation consisted of HANKS medium pH 7.4 with 10 μM DPI. The fluorescence signal was recorded at the excitation/emission wavelength pair of 488/525 nm using a fluorimeter (Hitachi, model F4500). Calibration was performed with known concentrations of dichlorofluorescein (DCF), which is the product of H_2_-DCF oxidation.

### Flow cytometry analysis of ROS release and cell death

Samples were analyzed in a FACSCalibur flow cytometer (BD Biosciences, San Jose, CA, USA) equipped with an argon laser and CellQuest software (version 4.1). Seven to ten thousand events were acquired for each sample. Spleen mononuclear cell populations were identified according to their light-scattering characteristics and enclosed in electronic gates, and the intensity of the fluorescent probe signal was analyzed. Superoxide (O_2_^•-^) detection was estimated by measuring the oxidation of dihydroethidium (DHE), as previously described [17]. Briefly, spleen mononuclear cells (10^6^ cells.mL^-1^) were incubated in RPMI 1640 medium containing 10% WT or HTG serum at 37°C in a humidified CO_2_ incubator (5% CO_2_) for 4 h. Next, cells were incubated with 5 μM DHE for 45 min under the same conditions and analyzed in the flow cytometer (FL2 channel). To estimate cell death, cells were suspended in binding buffer (10 mM HEPES pH 7.4, 150 mM NaCl, 5 mM KCl, 1 mM MgCl_2_ and 1.8 mM CaCl_2_) containing annexin V-FITC (1:500, Invitrogen, USA) and 7-amino-actinomycin D (7-AAD, 20 μg/μL, Molecular Probes, USA). Apoptosis was quantified as the number of annexin V-FITC-positive (FL1 channel) and 7-AAD-negative cells (FL2 channel), and necrosis was quantified as the number of 7-AAD-positive and annexin V-FITC-negative cells.

### Concentration of Interleukin-2

The concentration of endogenous interleukin-2 (IL-2) in spleen mononuclear cells was measured using the Endogen Mouse Interleukin-2 ELISA Kit (eBioscience), according to the manufacturer’s recommended procedure. Absorbance at 370 nm was determined using a microplate reader (Labsystems Multiskan MS type 352, Helsinki, Finland).

### Measurement of cytosolic free Ca^2+^ concentrations

Cells (15 × 10^6^ cells/mL) were loaded with 5 μM Fura-2 AM, a dual-wavelength radiometric indicator, in incubation medium containing 20 mM HEPES-Na buffer, pH 7.4, 120 mM NaCl, 3 mM KCl, 0.5 mM KH_2_PO_4_, 5 mM NaHCO_3_, 1.2 mM Na_2_SO_4_, 10 mM glucose, 1.2 mM MgCl_2_, 1.3 mM CaCl_2_ and 30 μg/mL BSA for 40 min. The cytosolic free Ca^2+^ concentration in Fura-2-loaded lymphocytes was determined at the excitation wavelengths of 340 and 380 nm and an emission wavelength of 510 nm using a fluorimeter (Hitachi, model F4500). Calibration was performed at the end of each experiment. [Ca^2+^]_cyt_ was calculated using the dissociation constant (Kd) of 225 nM for Ca^2+^-Fura-2.

### Detecting the mitochondrial cytochrome c released by Western blotting

Mouse liver subcellular fractions were obtained using the ProteoExtract Subcellular Proteome Extraction Kit, as described by the manufacturer’s instructions (Calbiochem, Darmstadt, Germany). The protein concentrations of mitochondrial and cytosolic extracts were quantified according to Bradford, 1976 [59]. Mitochondrial (5 μg protein) and cytosolic (10 μg protein) fractions were resolved by 12% SDS-PAGE and transferred onto a polyvinyldiene difluoride membrane. The membrane was blocked in 5% free fat free milk for 1 h and then incubated with anti-cytochrome c primary antibody overnight at 4°C, followed by incubation with HRP-conjugated secondary antibody for 1 h at room temperature. The immune-reactive bands were visualized using a chemiluminescence detection kit (GE Health Care, Piscataway, NJ, USA). The purity of the cytosolic fraction was determined by the absence of a VDAC band.

### Caspase 8 assay

Caspase 8 activity was assayed by measuring the release of an AFC-fluorescent compound from the synthetic peptide substrate Ac-LETD-AFC, using a Sigma kit and following the manufacturer’s instructions (Sigma, Saint Louis, Missouri, USA). Cells (3 × 10^6^ cells.mL^-1^) were suspended in 0.2 ml of chilled cell lysis buffer (20 mM HEPES pH 7.5, 10 mM KCl, 250 mM sucrose, 2 mM MgCl_2_ and 1 mM EDTA) containing 0.5 mM DTT. The cell suspension was sonicated (Misonix Sonicator S-3000, New Highway Farmingdale, New York, USA) for 3 cycles of 10 sec on ice at power setting 1; after that, cells were frozen at −80°C. The cell lysates were then thawed and centrifuged at 15,000 × *g* for 30 minutes. The supernatants were added to 0.2 mL of reaction buffer (i.e., 25 mM HEPES pH 7.5, 10% sucrose, 0.1% CHAPS) containing 10 mM DTT. Caspase 8 reactions were then initiated by the addition of 0.1 mM Ac-LETD-AFC and incubated for 1.5 h at 37°C. Fluorescence was measured using a Hitachi F4500 spectrofluorometer with excitation and emission wavelengths of 400 nm and 505 nm, respectively, and a slit width of 5.0 nm. Calibration was performed using free AFC (Sigma), which is the product of cleaved peptide substrate.

### Cell counts

One milliliter of blood was obtained from the retro-orbital plexus of anesthetized mice and stored in tubes containing 0.02 mL of the 7.5% ethylenediaminetetraacetic acid (EDTA). Complete blood counts were taken within an hour using a CELL-DYN 3700 SL analyzer (Abbott Diagnostics, Chicago, USA).

### Statistical analyses

Statistical analysis was performed using a one-way analysis of variance for multiple comparisons with Tukey’s post-hoc analysis (Figures 1, 2, 3, 5 and 6) and Student t-tests (Figure 4 and Table 1). The level of significance was set at P < 0.05. All data were analyzed using GraphPad Prism software, version 5 (GraphPad Software, USA).

## Abbreviations

DCF: Dichlorofluorescein; DHE: Dihydroethidium; DPI: Diphenyleneiodonium; EDTA: Ethylenediaminetetraacetic acid; FFA: Free fatty acids; GLY: Glyburide; HTG: Hypertriglyceridemic mice; H2DCF-DA: H_2_-dichlorofluorescein-diacetate; IL-2: Interleukin-2; mitoKATP: Mitochondrial ATP-sensitive potassium channel; ROS: Reactive oxygen species; PPARs: Peroxisome-proliferator-activated receptors; TG: Triglycerides; TLRs: Toll-like receptors; WT: Non-transgenic mice; 5-HD: 5-Hydroxidecanoate; 7-AAD: 7-Amino-actinomycin D.

## Competing interests

The authors declare that they have no competing interests.

## Authors’ contributions

The mice colony maintenance, plasma triglyceride levels, oxygen consumption, cytosolic free Ca^2+^, ROS production, caspase 8 activity and cell counts assays, as well as statistical analyses, were performed by LCA. Cytochrome c release was performed by CRP and interleukin-2 was performed by SRM. Flow cytometry analyses were performed by BAP and KGZ. LCA, RFC, AEV and HCFO designed research, analyzed data, wrote the paper and have primary responsibility for final content. All authors read and approved the final manuscript.
